# Haplotype approach for association analysis on hypertension

**DOI:** 10.1186/1753-6561-8-S1-S57

**Published:** 2014-06-17

**Authors:** Xiaowei Shen, Osvaldo Espin-Garcia, Xin Qiu, Yonathan Brhane, Geoffrey Liu, Wei Xu

**Affiliations:** 1 Department of Biostatistics, Princess Margaret Cancer Centre, 610 University Avenue, Toronto, Ontario, Canada M5G 2M9; 2 Department of Statistics and Actuarial Science, University of Waterloo, 200 University Avenue West, Waterloo, Ontario, Canada N2L 4G1; 3 Samuel Lunenfeld Research Institute, Mount Sinai Hospital, 60 Murray Street, Toronto, Ontario, Canada M5T 3L9; 4 Ontario Cancer Institute/Princess Margaret Cancer Centre, 610 University Avenue, Toronto, Ontario, Canada M5G 2M9; 5 Dalla Lana School of Public Health, University of Toronto, 155 College Street, Toronto, Ontario, Canada M5T 3M7

## Abstract

We applied a gene-based haplotype approach for the genome-wide association analysis on hypertension using Genetic Analysis Workshop 18 data for unrelated individuals. Association of single-nucleotide polymorphisms and clinical outcome were first assessed and haplotypes were then constructed based on the gene information and the linkage disequilibrium plot. Extensive haplotype analysis was also conducted for the whole chromosome 3. We found 1 block from the *ULK4 *gene and 2 blocks from the *LOC64690 *gene that were significantly associated with hypertension.

## Background

Hypertension is a major risk factor for many diseases, including stroke and heart failure. Various genetic studies have been done and a number of genes have been identified as having strong associations with hypertension or high blood pressure [1]. In our study, we proposed a haplotype approach to identify blocks on the gene that have strong associations with hypertension. Focusing on a block of the gene instead of looking only at a particular point may better capture the disease pattern and take the potential interactions between markers into account [2]. In addition, because the number of tests is reduced compared with the single-nucleotide polymorphism (SNP) tests, there is less penalty from multiple testing [3]. We report significant haplotypes from association analysis.

## Methods

### Definition of outcome and predictors

Hypertension was defined as systolic blood pressure >140 mm Hg and diastolic blood pressure > 90 mm Hg, or as being on antihypertensive medications at a specific examination. For this study, we defined our outcome as "ever-hypertension" if an individual was hypertensive in any of the 4 examinations, and "never-hypertension" if hypertension criteria were never met in those 4 examinations. In this way, we created a single hypertension outcome based on the longitudinal structure of the data. The genetic analysis was focused on unrelated individuals.

**Figure 1:**
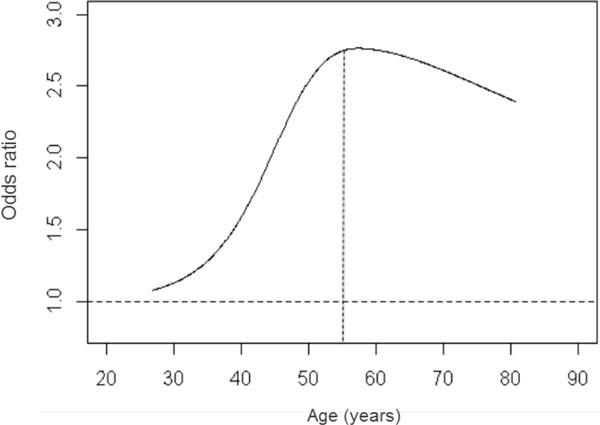
Cubic splines plot for age

Gender, smoking habits, and age were selected as the main clinical predictors based on exploratory data analysis. Similar to the definition of outcome, smoking was defined as "ever-smokers" and "nonsmokers" based on multiple examinations. We first treated age as a continuous variable and detected its significant association with hypertension (odds ratio [OR] = 1.034; 95% confidence interval [CI]: 1.009, 1.059; *p *value = 0.0075). Then we examined the possible nonlinear relation between age and the defined hypertension outcome based on restricted cubic spline method [4] and found that the pattern of OR changed as age changed. Finally, based on the cubic splines plot (Figure 1), we dichotomized age at 55 years.

### Quality control of genotype data

**Figure 2:**
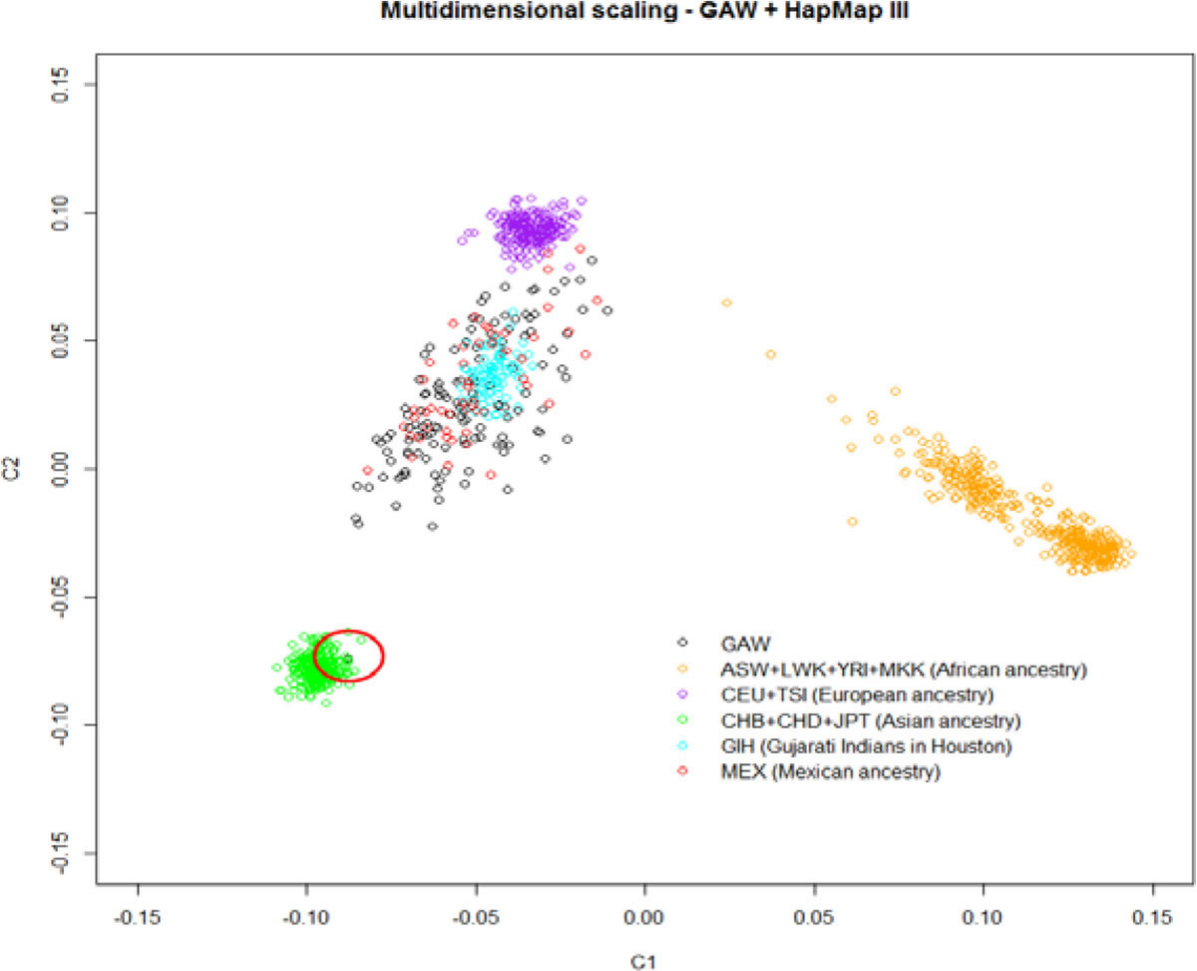
Multidimensional scaling plot (outlier in red circle)

We focused on genome-wide association studies data of chromosome 3, and conducted quality control of genotype data using PLINK [5]. Thresholds for data quality control steps were set as follows: individual genotyping missing rate at 0.05, minor allele frequency at 0.1, missing rate per SNP at 0.05, and Hardy-Weinberg equilibrium at 1 × 10^−6^. Heterozygosity rate was assessed for potential outliers. We merged our data set with HapMap [6] data and generated a multidimensional scaling plot (Figure 2). To adjust for population stratification effect, we used EIGENSTRAT [7,8] to conduct principal components analysis to explicitly model ancestry differences between individuals and obtained a principal component for each subject.

### Preliminary analysis and gene-based haplotype construction

A logistic regression model was applied on association analysis for SNPs and the defined hypertension outcome with adjustment for covariates as well as principal component vectors obtained from the population stratification procedure. We first found some nominally significant SNPs (*p *<5 × 10^−4^) from this preliminary model, and then located the genes corresponding to such SNPs based on the annotation information (T. Nalpathamkalam et al., unpublished data, 2012). For each gene, we defined the haplotype block based on a high linkage disequilibrium (LD) region containing the significant SNPs we found from the preliminary model. The blocks were defined by CI algorithm [9] as well as the 4-gamete rule algorithm [10]. Then for each block, we estimated the haplotype frequencies and the probability of having each haplotype for all individuals. The estimations of the LD blocks and haplotype frequencies were applied using HAPLOVIEW [11] and PHASE [12-14].

### Haplotype analysis

First, omnibus tests on haplotypes were performed for each block of interest. Similar to the preliminary association analysis, logistic regression models were used and then likelihood ratio tests were conducted to see if haplotypes should be included in the model:

(1) $$logit\left(P\left(Y_{i}=1|\vec{X_{i}}\right)\right)=\beta_{0}+\beta_{1}X_{1i}+\beta_{2}X_{2i}+\beta_{3}X_{3i}+\beta_{4}X_{4i}+\beta_{5}X_{5i}$$

(2) $$logit\left(P\left(Y_{i}=1|\vec{X_{i}}\right)\right)=\beta_{0}+\beta_{2}X_{2i}+\beta_{3}X_{3i}+\beta_{4}X_{4i}+\beta_{5}X_{5i}$$

where *Y *represents outcome (*Y_i _*= 1 if individual *i *is defined as "ever-hypertension"), *X_1 _*the design matrix representing haplotypes in a particular block, *X_2 _*age, *X_3 _*gender, *X_4 _*smoking habit, and *X_5 _*principal component. Difference of log-likelihood between model (2) and model (1) were calculated and a chi-square test was performed. The entries in the design matrix *X_1 _*were the inferred conditional probabilities of haplotypes given the genotype [15]. Specifically, for haplotypes *h_m _*and *h_n_*, the conditional probability of the pair (*h_m_, h_n_*) for the *i*^th ^individual with genotype *G_i _*is:

(3) $$\text{Pr}\left(h_{m},h_{n}|G_{i}\right)=\frac{\text{Pr}\left(G_{i}\text{|}h_{m},h_{n}\right)p_{h_{m}}p_{h_{n}}}{\sum_{u,v}\text{Pr}\left(G_{i}\text{|}h_{u},h_{v}\right)p_{h_{u}}p_{h_{v}}}$$

where *p_hu _*and *p_hv _*denote haplotype frequencies estimated from PHASE. If the omnibus test was significant, which means at least 1 haplotype should be kept in the model, we then conducted haplotype-specific tests for each haplotype in the block and identified the specific haplotype strongly associated with the outcome.

## Results

### Summary of phenotypes and genotypes

We started with 65,460 SNPs of 142 unrelated individuals. First, we checked missing rate per individual at the 0.05 level and dropped 9 individuals. Second, we excluded SNPs with a minor allele frequency less than 0.1, leaving 46,205 SNPs in the sample. Following that, we excluded SNPs with missing rate greater than 0.05, leaving 46,103 SNPs. Finally, we checked the Hardy-Weinberg equilibrium at 1 × 10^−6 ^level, and all 46,103 SNPs passed the test. Heterozygosity rate was checked for all individuals and none were located outside ±3 SD from the mean heterozygosity rate. We then combined the cleaned data set with HapMap data on common SNPs and obtained the multidimensional scaling plot (see Figure 2). One outlier was identified from family 9 (T2DG0901244), who probably belonged to an Asian population. After quality control, we excluded this individual from the samples and ended up with 42,727 SNPs and 132 individuals. For the 132 individuals left in our sample, 81 were classified as "ever-hypertension" and 51 as "never-hypertension." Table 1 summarizes the distributions of covariates.

**Table 1:** Summary of phenotype data

Characteristics		Count (%)
Hypertension	Ever	81 (61.4)
	Never	51 (38.6)

Gender	Male	57 (43.2)
	Female	75 (56.8)

Smoking	Ever	32 (24.2)
	Never	100 (75.8)

Age	<55 years	75 (56.8)
	≥55 years	57 (43.2)

### Preliminary association analysis and haplotype construction

The preliminary model had limited power to detect SNPs that strongly associated with hypertension after multiple testing was adjusted. We used QUANTO [16] to conduct power analysis. We needed 433 individuals to have an 80% power to detect the marginal effect of OR = 2.0. Table 2 lists the top 8 SNPs from the preliminary model. They were from 5 genes that may have potential associations with hypertension. Haplotypes were constructed on these genes based on results from the LD plot generated by HAPLOVIEW, and then sample haplotype frequencies were estimated.

**Table 2:** Significant SNPs from preliminary model and corresponding genes

SNP	Gene	OR (CI)	*p *Value
rs2700464	*ULK4*	0.29 (0.15, 0.56)	2 × 10^−4^
rs2470696	*CBLB*	0.31 (0.18, 0.55)	7 × 10^−5^
rs2953768	*ALG1L2*	0.18 (0.08, 0.39)	2 × 10^−5^
rs6785346	*LOC64690*	3.53 (1.87, 6.64)	9 × 10^−5^
rs9857853	*LOC64690*	3.19 (1.74, 5.87)	2 × 10^−4^
rs9848025	*LOC64690*	3.52 (1.86, 6.66)	1 × 10^−4^
rs2129379	*LOC64690*	3.59 (1.77, 7.28)	4 × 10^−4^
rs16862964	*LPP-AS2*	4.95 (2.06, 11.89)	3 × 10^−4^

### Haplotype analysis

One haplotype from a candidate block of gene *ULK4 *had significant association with hypertension in the main effect model. Haplotypes from 2 blocks of gene *LOC64690 *were also significant in the main effect model. We took multiple testing into consideration and determined the significance threshold as 0.05/number of haplotypes being tested in the candidate block. Table 3 summarizes the results from the haplotype analysis. Age was significant in both models, but gender and smoking habit were not.

**Table 3:** Significant haplotypes from model 1 in "Methods: Haplotype analysis" section

Gene (SNP)	Covariate	OR (CI)	*p *Value	Haplotype Frequency
*ULK4 *(rs2700464)	TAAC	2.7215 (1.3998, 5.2912)	0.0032	0.3147
	Age	2.7489 (1.2476, 6.0569)	0.0121	

*LOC64690 *(rs6785346, rs9857853)	CC	0.2430 (0.1202, 0.4913)	1 × 10^−4^	0.6170
	Age	3.3028 (1.4293, 7.6320)	0.0052	

*LOC64690 *(rs9848025)	GCGTG	3.8169 (1.7371, 8.3867)	9 × 10^−4^	0.2477
	Age	3.6333 (1.5983, 8.2590)	0.0021	

Adding the interactive effect of haplotype and age did not improve the model. Power analysis showed that for gene *ULK4*, we needed at least 258 individuals to have an 80% power to detect interaction effect with ratio of OR = 2.0, but only 92 individuals were required for the main effects model. For gene *LOC64690*, 514 individuals were required to gain 80% power for the interaction model (given ratio of OR = 2.0), but only 100 individuals were required for the main effects model to achieve the same level of power.

We also conducted haplotype analysis on whole chromosome 3 in PLINK. In PLINK, haplotype blocks are estimated following the default procedure in HAPLOVIEW and pairwise LD is calculated only for SNPs within 100 kilobases (kb). We tried the models with and without adjusted covariates. A total of 6389 haplotype blocks were constructed by using PLINK and no haplotype was significant in the omnibus test at Bonferroni corrected significance level of 0.05/6389 ~ 8 × 10^−6^.

## Conclusions

Based on the results, we can see that the haplotype containing SNP rs2700464 on *ULK4 *is strongly associated with our defined hypertension outcome. Daniel et al [17] concluded that *ULK4 *is associated with high blood pressure and, potentially, hypertension. We also detected that 2 haplotype blocks on *LOC64690 *had a strong relationship with hypertension. In addition, the interaction effect between age and haplotype was not significant in all models, but power analysis indicated that our sample size was too limited to detect interaction effect, but sufficient for the main effects model.

We focused only on unrelated individuals in our study, ignoring family structures. We may consider including the family structure in further research, and may try to model the complex relationship between family members. In addition, we ran the permutation test for haplotypes in the candidate blocks as well as on the whole chromosome 3. However, the population structure is not preserved for a logistic model when doing permutation tests. Therefore, the permutation *p *values may not be a good estimate of the asymptotic *p *values. We may consider using the biased urn method [18] to overcome this problem in further research.

## Competing interests

The authors declare that they have no competing interests.

## Authors' contributions

XS and WX designed the overall study. XS conducted statistical analyses and drafted the manuscript. OEG, XQ, YB, and GL conceived of the study, participated in its design and coordination, and helped to draft the manuscript. All authors read and approved the final manuscript.
